# Supplementary material for: Causal Relationship between Aging and Anorexia Nervosa: A White-Matter-Microstructure-Mediated Mendelian Randomization Analysis
Source: Biomedicines. 2024 Aug 16;12(8):1874. doi: 10.3390/biomedicines12081874 (PMC11351342; doi:10.3390/biomedicines12081874)
Supplement: Supplementary file 1 [file biomedicines-12-01874-s001.zip › Supplementary Figure/Figure S4/Singe SNP plot of MR analysis for TL to ubm-b-1656.pdf]

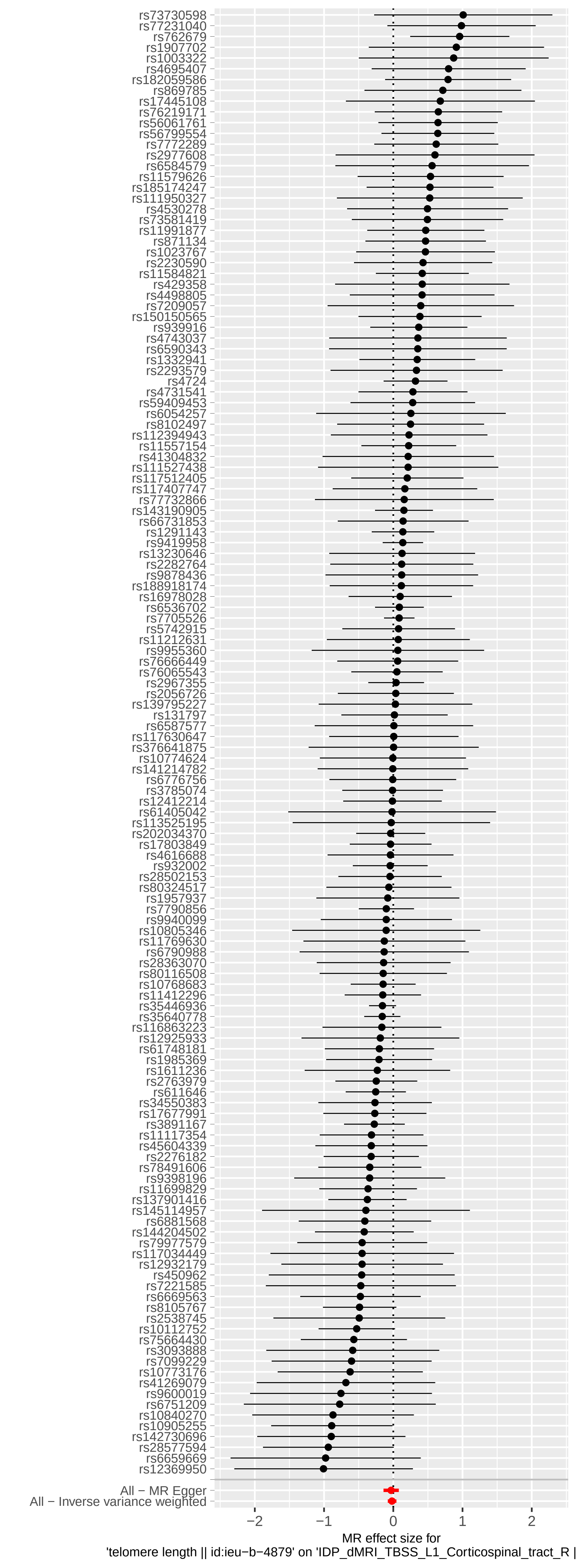
rs73730598
rs77231040
rs762679
rs1907702
rs1003322
rs4695407
rs182059586
rs869785
rs17445108
rs76219171
rs56061761
rs56799554
rs7772289
rs2977608
rs6584579
rs11579626
rs185174247
rs111950327
rs4530278
rs73581419
rs11991877
rs871134
rs1023767
rs2230590
rs11584821
rs429358
rs4498805
rs7209057
rs150150565
rs939916
rs4743037
rs6590343
rs1332941
rs2293579
rs4724
rs4731541
rs59409453
rs6054257
rs8102497
rs112394943
rs11557154
rs41304832
rs111527438
rs117512405
rs117407747
rs77732866
rs143190905
rs66731853
rs1291143
rs9419958
rs13230646
rs2282764
rs9878436
rs188918174
rs16978028
rs6536702
rs7705526
rs5742915
rs11212631
rs9955360
rs76666449
rs76065543
rs2967355
rs2056726
rs139795227
rs131797
rs6587577
rs117630647
rs376641875
rs10774624
rs141214782
rs6776756
rs3785074
rs12412214
rs61405042
rs113525195
rs202034370
rs17803849
rs4616688
rs932002
rs28502153
rs80324517
rs1957937
rs7790856
rs9940099
rs10805346
rs11769630
rs6790988
rs28363070
rs80116508
rs10768683
rs11412296
rs35446936
rs35640778
rs116863223
rs12925933
rs61748181
rs1985369
rs1611236
rs2763979
rs611646
rs34550383
rs17677991
rs3891167
rs11117354
rs45604339
rs2276182
rs78491606
rs9398196
rs11699829
rs137901416
rs145114957
rs6881568
rs144204502
rs79977579
rs117034449
rs12932179
rs450962
rs7221585
rs6669563
rs8105767
rs2538745
rs10112752
rs75664430
rs3093888
rs7099229
rs10773176
rs41269079
rs9600019
rs6751209
rs10840270
rs10905255
rs142730696
rs28577594
rs6659669
rs12369950
All − MR Egger
All − Inverse variance weighted
−2
−1
0
1
2
MR effect size for
'telomere length || id:ieu−b−4879' on 'IDP_dMRI_TBSS_L1_Corticospinal_tract_R |
